# Cyriax Friction Massage—Suggestions for Improvements

**DOI:** 10.3390/medicina55050185

**Published:** 2019-05-21

**Authors:** Alexios Pitsillides, Dimitrios Stasinopoulos

**Affiliations:** 1 Department of Health Sciences, School of Sciences, European University Cyprus, Nicosia 1516, Cyprus; alexpitsillides@gmail.com; 2 Director of Cyprus Musculoskeletal and Sports Trauma Research Centre (CYMUSTREC), Nicosia 1516, Cyprus

**Keywords:** Cyriax method, transverse frictions, deep friction massage, deep transverse massage, friction massage

## Abstract

*Background and objectives:* Cyriax friction massage is a widely known and used technique in the field of chronic pain management. Despite its frequent use in daily clinical practice, the technique lacks evidence to support its therapeutic value. While this might be due to various factors, the authors of this paper suggest that the technique might need to be improved and/or modernized according to the recent literature. The purpose of this letter is to further analyze our point of view. *Materials and Methods:* Using the most relevant methods to the subject literature, the authors intended to point out a few technical details that might need reconsideration and/or modernization. *Results:* An appropriate terminology is suggested in the text. Further, suggestions are made regarding the technique’s interval time, a possible addition of self-treatment, a discussion of the combination with Mill’s manipulation, tendon positioning and other parameters. *Conclusions:* As a therapeutic value has not yet been clearly documented, and since the modernization and/or improvement of the technique might be needed, we suggest that this technique should not be used as a first-line treatment for the management of chronic pain.

## 1. Introduction

Deep friction massage (DFM) has been promoted and popularized by Cyriax to the clinical world, even though Mennell first suggested the use of specific massage movements called frictions [1,2]. To date, the efficiency of the technique has not been documented, despite the fact that DFM is a widely known technique. Relevant reviews have been hampered by deterrent factors such as sample sizes as well as several methodological limitations of the included studies, such as the lack of standardization of the DFM protocol [3,4,5]. We believe that is indeed a possibility that the therapeutic results of the method have not been documented yet due to the factors found by the authors of the previously mentioned reviews. On the other hand, we suspect that the inability to record the therapeutic results of DFM might be due to technical faults of the instructions proposed by Cyriax. 

## 2. Terminology

Incorrect terminology may have negative effects on the results of the technique, as it appears to affect how it is executed [6]. Τhis observation has been made by other researchers as well [7], but a proposal for an appropriate terminology cannot be found in the published literature as far as we know. The only discrimination we could find is the one between an acute and a chronic injury in the latest edition of Cyriax’s book, but this only describes the grade of the technique that has to be applied [6]. Considering firstly that the correct application depends on proper friction intensity, duration and frequency, and secondly that nonspecific terms might be a deterrent factor, we believe that setting specific terms might influence clinical results. 

In our opinion, the general term of the technique should be transverse friction massage (TFM). We believe that previously suggested terms are not completely correct. Any term that includes the word “deep” should not be used. We believe that any term with this characteristic cannot include the application that has to be done for an acute injury, since it will have to be mostly superficial to the tissue, and only six sweeps should be performed on the target tissue [6]. The word “friction” should be included in our opinion. Even though, in an acute stage, the friction on the target tissue is limited to six sweeps, we suggest that the word “friction” should be part of the general terminology of the technique. Our suggestion comes from the fact that friction on the target tissue is the aim of the technique, and thus this has to be clear in the general terminology. The term “massage” has to be included as well. In this way, a new reader will understand that that technique does not only involve friction. Thus, in an acute phase, transverse massage is applied until analgesia and from that point, once target tissue is reached, six sweeps (frictions) more. Additionally, as in chronic injuries transverse massage is applied until analgesia, and from that point 10 minutes of friction is applied to the target tissue, we suggest as a general term the one mentioned above, transverse friction massage (TFM).

In an acute injury, we suggest the term gentle transverse massage (GTM). The differentiation from the term suggested in Cyriax’s book is the word “massage” instead of “friction”. We believe massage is a better term to describe the procedure performed at an acute stage, which is a gentle transverse massage of the area until analgesia and then six sweeps more. 

On the other hand, we are in agreement with the suggestion made in the book for chronic injuries. We support the term deep transverse friction (DTF) since it is what is mostly performed in this situation. 

## 3. Interval between Sessions

We believe that the instructions concerning the interval between interventions in a chronic stage are incorrectly left to the discretion of the therapist. While a comparison of different intervals between TFM sessions does not exist, the instruction of a minimum 48 h [6] is not enough in our opinion. We believe that basic biological differences between the tissues that the technique could be applied to are ignored. 

Specifically, oxygen consumption by tendons and ligaments is 7.5 times lower than skeletal muscles. Given their low metabolic rate and well-developed anaerobic energy generation capacity, tendons are able to carry loads and maintain tension for long periods while avoiding the risk of ischemia and subsequent necrosis. However, a low metabolic rate results in slow healing after injury [8]. Thus, we believe that guidelines should be suggested according to the target tissue. We suggest that tendons and ligaments are treated every third day, securing a balance between collagen synthesis and degradation [9]. Another reason that leads to this suggestion is the slower healing after micro injuries due to the technique, due to the aforementioned biological differences. We believe tendons and ligaments should be treated every third day due to the fact that type I collagen’s response to high load in a normal tendon peaks at around 3 days after intense loading [10,11,12]. This response to load appears to be greater in pathological tendons than normal tendons, with a lower resting level and elevated response to loading [10]. We believe that, during the interval time, the tendon should still be somehow loaded to favor the healing process. A possible way to follow this is suggested and further analyzed in the following paragraph. It is also important to mention that the biological tendon response to TFM on humans has not been reported yet, and our suggestions come as a result of studies investigating changes induced by exercise [9,11,12].

## 4. Self-Treatment

We believe that the technique as proposed by Cyriax does not include the element of self-treatment. It is in our opinion impossible to compare a method that loads the affected tissue for a minimum of 10 min every 48 h (current Cyriax suggestion) to any other technique that loads the same tissue in a more logically structured way [13]. In such a comparison, the results might not support TFM. This might be due to the loading frequency and not the efficiency of the technique itself, since the healing process will be favored by light loading in the interval between sessions. By concluding our thinking, we believe that educating the patient to self-execute GTM between sessions may improve TFM results and offer more equality in the comparison of the technique with others. Training the patient to execute GTM might be beneficial in an acute injury. Since the analgesic effect of TFM can last from 0.3 min to 48 h [14], the patient will be given a way to control their pain, at least for those in which the analgesic duration would have been below the mean (24 h). Unfortunately, only one previous study [14] has examined the duration of the TFM analgesic effect. Due to this lack of evidence, we are unable to recommend a possible dosage of GTM to control pain.

With all this in mind, we suggest that the patient should be taught to control their pain by gently massaging the affected area only until analgesia. In this way, in the case of an acute injury, better pain control will be offered. In the case of a chronic injury, a moderated load will be applied on the tissue in the interval between sessions, positively affecting the new tissue produced by the caused micro injuries during the physiotherapy session, aiding the healing process. 

## 5. Mill’s Manipulation for Lateral Elbow Tendinopathy (LET)

In our opinion, Cyriax’s suggestion that TFM should always be followed by Mill’s manipulation when faced with lateral elbow tendinopathy (LET) is wrong and restrictive for the results of the method. The only reason that the technique is followed by this specific manipulation in the case of LET is because of Cyriax’s suggestion [15]. With no evidence supporting the idea that the combination of the technique with Mill’s manipulation is superior to a combination with another manipulation or TFM alone [15], we believe that such a restriction is anecdotal.

It is our belief that LET should be either treated similarly to other tendinopathies, or that specific manipulations should be combined in every tendinopathy treatment. To answer this, well-designed RCTs should be designed.

## 6. Tendon Position

Another point that we believe needs modernization is the position of the tendon during the application of the technique. In our opinion, tendons should be placed in a stretched position, which should be determined by the perception of discomfort and not pain. It has been shown that when the muscle–tendon unit is stretched, most of the stretching occurs in the muscle [16]. A percentage of the force exerted on the tendon during the TFM is possibly absorbed by the muscle. A greater stretch, in our opinion, might be translated into better therapeutic results, as it would correspond to a higher probability of the alignment of the tendon fibers. 

## 7. Other Parameters

As described by Cyriax [6], the guidelines are largely based on clinical opinion. In terms of application duration and sessions, we believe as much as necessary should be used to produce the required therapeutic results. In order to suggest a proper treatment time and number of sessions, we believe that relative RCTs should be designed. 

It is widely known and supported by evidence that treating a musculoskeletal disorder is not simply about breaking adhesions and promoting collagen alignment (if this effect is indeed produced by TFM). A good rehabilitation should also address specific tissue characteristics. In the example of tendons, energy storage and release are essential and vital [7]. TFM does not affect this parameter, and so we disagree with Cyriax that TFM combined with manual therapy can efficiently rehabilitate a tendon. Another tissue-specific parameter that is not addressed by TFM is tendon neuroplasticity [17].

We believe that, at the moment, TFM is not supported by sufficient evidence, which creates difficulties for proper parameters to be suggested. This might be a reason that the technique’s efficacy has not yet been shown [1,2]. It is also a possibility this is the origin of the non-compliance with Cyriax’s protocol in clinical practice, where physiotherapists undertake their clinical reasoning based on the patient’s specific needs, adjusting the recommended dose to their interpretation of the clinical condition [1,2].

We believe that there is a reasonable theoretical background behind the theory of TFM, but that it needs modernization and further investigation. A better application of the technique will be translated not only to a better management of pain, but also to a better rehabilitation of musculoskeletal injuries.

## 8. Conclusions

While there is not sufficient evidence regarding the efficacy of TFM, it is our personal opinion that it should not be in the first line of musculoskeletal treatments.
